# Strong Host-Feeding Preferences of the Vector *Triatoma infestans* Modified by Vector Density: Implications for the Epidemiology of Chagas Disease

**DOI:** 10.1371/journal.pntd.0000447

**Published:** 2009-05-26

**Authors:** Ricardo E. Gürtler, Leonardo A. Ceballos, Paula Ordóñez-Krasnowski, Leonardo A. Lanati, Raúl Stariolo, Uriel Kitron

**Affiliations:** 1 Laboratory of Eco-Epidemiology, Department of Ecology, Genetics and Evolution, Universidad de Buenos Aires, Buenos Aires, Argentina; 2 National Vector Control Coordination, Center for Chagas Disease Reservoirs and Vectors, Córdoba, Argentina; 3 Department of Environmental Studies, Emory University, Atlanta, Georgia, United States of America; National Institute of Allergy and Infectious Diseases, United States of America

## Abstract

**Background:**

Understanding the factors that affect the host-feeding preferences of triatomine bugs is crucial for estimating transmission risks and predicting the effects of control tactics targeting domestic animals. We tested whether *Triatoma infestans* bugs prefer to feed on dogs *vs.* chickens and on dogs *vs.* cats and whether vector density modified host choices and other vital rates under natural conditions.

**Methodology:**

Two host choice experiments were conducted in small caged huts with two rooms between which bugs could move freely. Matched pairs of dog–chicken (six) and dog–cat (three) were assigned randomly to two levels of vector abundance and exposed to starved bugs during three nights. Bloodmeals from 1,160 bugs were tested by a direct enzyme-linked immunosorbent assay.

**Principal Findings:**

Conditional logistic regression showed that dogs were highly preferred over chickens or cats and that vector density modified host-feeding choices. The relative risk of a bug being blood-engorged increased significantly when it fed only on dog rather than chicken or cat. Bugs achieved higher post-exposure weight at higher vector densities and successive occasions, more so if they fed on a dog rather than on a cat.

**Conclusions:**

Our findings strongly refute the hypothesis that *T. infestans* prefers to blood-feed on chickens rather than dogs. An increase in dog or cat availability or accessibility will increase the rate of bug feeding on them and exert strong non-linear effects on *R*_0_. When combined with between-dog heterogeneities in exposure, infection, and infectiousness, the strong bug preference for dogs can be exploited to target dogs in general, and even the specific individuals that account for most of the risk, with topical lotions or insecticide-impregnated collars to turn them into baited lethal traps or use them as transmission or infestation sentinels based on their immune response to *Trypanosoma cruzi* or bug salivary antigens.

## Author Summary

Chagas disease is a complex zoonosis with more than 150 mammalian host species, nearly a dozen blood-sucking triatomine species as main vectors, and 9–11 million people infected with *Trypanosoma cruzi* (its causal agent) in the Americas. *Triatoma infestans*, a highly domesticated species and one of the main vectors, feeds more often on domestic animals than on humans in northern Argentina. The question of whether there are host-feeding preferences among dogs, cats, and chickens is crucial to estimating transmission risks and predicting the effects of control tactics targeting them. This article reports the first host choice experiments of triatomine bugs conducted in small huts under natural conditions. The results demonstrate that *T. infestans* consistently preferred dogs to chickens or cats, with host shifts occurring more frequently at higher vector densities. Combined with earlier findings showing that dogs have high infection rates, are highly infectious, and have high contact rates with humans and domestic bugs, our results reinforce the role of dogs as the key reservoirs of *T. cruzi*. The strong bug preference for dogs can be exploited to target dogs with topical lotions or insecticide-impregnated collars to turn them into baited lethal traps or use them as transmission or infestation sentinels.

## Introduction

Host choice of hematophagous insects mainly depends on relative host abundance and proximity, host defensive behavior, the density of blood-sucking insects, and the spatial and temporal concurrence of hosts and insects [1],[2]. Examples of innate (genetically determined) host-feeding preferences are few, and convincing evidence with both experimental and field support is scarce [1],[3]. Fleas (*Xenopsylla conformis*) do not have an innate preference but can discriminate between juvenile and adult hosts, and derive a higher reproductive reward when feeding on juvenile hosts [4]. In *Lutzomyia longipalpis* sandflies, host size was the main determinant of host-feeding choices among a human, a dog and a chicken exposed simultaneously to laboratory-reared sandflies [5], and its feeding success on chickens was density-dependent [6]. For *Triatoma infestans* bugs [7] and *Simulium damnosum* blackflies [8], the proportion of insects biting humans was strongly density-dependent. For *Glossina palpalis gambiensis* tsetse flies, male flies preferred to feed on cattle rather on reptiles in a stable; the host species selected for the second bloodmeal depended on the host encountered for the first bloodmeal, the between-meal interval and the interaction between these two factors [9]. In mosquitoes, acquired feeding preferences are reflected in their tendency to return to the same villages, houses, host species and oviposition sites [10]. A non-homogeneous distribution of vector feeding contacts on the same host species leads to a basic reproduction number of the pathogen (*R*_0_) greater than or equal to that obtained under uniform host selection, a result that still holds when groups of mosquitoes and hosts are highly structured in patches [11],[12].

Triatomine bugs (Hemiptera: Reduviidae) are the vectors of *Trypanosoma cruzi*, the causal agent of Chagas disease. *Triatoma infestans* (Klug), the main vector of *T. cruzi*, is a highly domiciliated species that also occurs in peridomestic structures housing domestic animals [13]. Like most species of triatomine bugs, *T. infestans* shows eclectic host-feeding patterns [14],[15]. Host proximity has usually been considered more important than host preference for hungry bugs seeking to feed [14]. In laboratory-based host choice experiments of *Triatoma sordida* (a species typically associated with birds), first-instar nymphs significantly preferred birds to humans [16] whereas fifth-instar nymphs feeding success and bloodmeal size were significantly larger on guinea pigs than on pigeons [17]. *Triatoma infestans* preferred caged chickens to guinea pigs though not in all replicates [18]. In a simultaneous exposure of four caged vertebrate species to separate groups of fifth-instar nymphs of *T. infestans*, *Triatoma dimidiata* and *Rhodnius prolixus*, none displayed dominant host-feeding preferences among dogs, chickens and opossums but toads were only rarely fed upon [19]. These authors [19] concluded that *T. infestans* showed a slight preference for dogs in short daytime experiments and a slight one for chickens in overnight trials. No measure of variability in host-feeding choices between the 7–22 replicates for each triatomine species was reported and neither were statistical procedures described. Within the restricted experimental conditions used, the tested triatomine species do not appear to have a fixed or dominant preference for any of the study hosts, and the question whether there are host-feeding preferences between dogs and chickens is still unresolved.

In rural areas of the Argentine Chaco, domestic *T. infestans* blood-fed more frequently on dogs or chickens than on the human hosts or cats with which they shared sleeping quarters [7]. Seasonal host shifts were recorded. In spring-summer bug collections, the proportion of domestic bugs that fed on dogs increased significantly with increasing numbers of dogs and *T. infestans* in bedroom areas, and decreased as bug feeding frequency on chickens rose. Feedings on cats increased significantly with the number of cats and decreased with the number of dogs in bedroom areas. Dog-fed *T. infestans* had higher infection prevalence with *T. cruzi* than bugs feeding on other hosts, but many bugs within a given house fed on up to four different bloodmeal sources in summer [20]. Both domestic dogs and cats acted as a source of *T. cruzi* infection to other species, including humans, whereas chickens (not susceptible to *T. cruzi*) contributed strongly to bug population growth [21],[22]. Using molecular typing techniques of *T. cruzi*, we recently showed that dogs, cats and a large fraction of the *T. infestans* within a household shared the same parasite sublineage and therefore were connected epidemiologically [23].

Understanding the factors that affect the host-feeding selection patterns of triatomine bugs is crucial to estimating transmission risks and predicting the putative effects of introducing or removing domestic animal hosts or targeting them for control. The recent emergence of pyrethroid resistance in *T. infestans* in northern Argentina and Bolivia [24], combined with the low effectiveness of standard residual spraying of pyrethroid insecticides in peridomestic structures [25], gave strong impetus to the search for cost-effective, alternative treatments based on the application of powder, topical lotions or insecticide-impregnated collars to the domestic animals themselves [26]–[28]. Whether chickens or dogs would be the preferred targets is one of the questions that motivated the current study, in which we report the first binary discrete host choice experiments of triatomine bugs conducted in small mud-and-thatch huts under natural climatic conditions. We tested whether *T. infestans* displayed host-feeding preferences between dogs and chickens and between dogs and cats, all unrestrained, and whether the density of vectors per hut modified host-feeding success, blood-engorgement and other vital rates in replicated trials. Analysis of field blood-feeding patterns and laboratory experiments supported the hypothesis that *T. infestans* would prefer chickens to dogs and dogs to cats, though the evidence regarding chickens and dogs was inconclusive [15],[19],[29]. We also hypothesized that blood-feeding success, engorgement and post-exposure bug weight would be reduced in a density-dependent way [13],[30]. To infer the putative processes accounting for the observed discrepancies, we re-examined the reported host-feeding patterns of domestic *T. infestans* in the field in light of experimental host choices and the demographic and behavior patterns of domestic animal hosts.

## Materials and Methods

### Study Site

The trials were carried out in the field station run by the Argentinean National Vector Control Program in Punilla, Province of Córdoba (31°14′S, 64°28′W) in summer (late January) and in early winter (June) 2006. Study location and experimental set-up were previously described [27],[31]. For the present study, six small experimental huts simulating typical mud-and-thatch houses (1.60×0.80×0.80 m with a 40 cm-wide entrance) were built and subdivided into two equally-sized rooms that shared an adobe-bricked wall with loose bricks; this arrangement allowed the bugs to hide and move freely between rooms. The lower third of the middle wall and all of the other walls were plastered on the inside with a 7∶1 mixture of soil and cement, and a cement carpet was added over the floors of beaten earth. A cage of plastic mosquito netting mounted on a metal frame was placed above each hut to prevent bugs from escaping. The six huts were arranged in two rows over a 50 m^2^ rectangle.

### Animals

Seven mongrel male dogs (approximate age range, 4–7 years; mean weight, 10.8 kg; SD, 3.4; range, 7–15) were used in the trial. All dogs had been exposed to *T. infestans* and had worn deltamethrin-impregnated collars for a four-month period ending six months before the current experiment [27] but not thereafter. According to the collars' manufacturer, the residual effect of the insecticide should cease within six months or one month after removing the collars; since collar use started >10 months before the first trial, no residual effect was expected to occur at this time. Dogs were vaccinated and dewormed with mebendazole prior to the start of the trial; they were kept in kennels made of chicken wire and a roof and fed twice daily. Chickens (all females; approximate age range, 2–2.5 years; mean weight, 2.4 kg; SD, 0.3; range, 1.9–2.8) of Lohmann breed were identified with a color ribbon and kept separately from other animals in a similar pen. Cats were a female and two male adults (approximate age range, 2.5–4 years; mean weight, 2.7 kg; SD, 0.2; range, 2.5–2.9). Dogs, but not chickens or cats, had previously been exposed to *T. infestans* bites six months before the first trial [27]. Chickens and cats had not been treated with insecticides. During the trials each animal was stationed individually inside a specific experimental hut at sunset and then released every morning into its specific area within the compound. This study complied with guidelines on research and biological testing activities involving live vertebrate animals from the Institutional Animal Care and Use Committee (IACUC) at FCEN-UBA, which is based on the International Guiding Principles for Biomedical Research Involving Animals developed by the Council for International Organizations of Medical Sciences.

The *T. infestans* bugs used in these experiments were first or third generation from bugs collected in Córdoba, Santiago del Estero and San Luis (Argentina); they had been reared on chickens at the insectary (at 27°C, relative humidity 70%), fed to repletion on the fourth instar, and starved for 2.5 (first trial) to 3.5 months (second trial) prior exposure to the hosts. This long starvation period (>2 months after they molted to fifth instars) normally does not increase bug mortality, and was used to secure that the previous bloodmeals on chickens were completely digested at the time of the trials (i.e., no ‘false positive” bloodmeal). Before release, a 20% sample of triatomine bugs for each trial was weighed individually with an electronic balance (precision, 0.1 mg), and the volume and shape of the bugs' midgut was observed by transparency against a torch light to check their nutritional status semi-qualitatively based on the size of the bug abdomen and occurrence of blood remnants [27],[32]. This classification (by which bugs are scored as unfed, little fed, medium fed, and fully fed) was consistent between observers. All bugs were classified as unfed immediately before the trials.

### Study Design

The first trial was started on late January 2006 (summer) and included six matched dog-chicken pairs, each housed in a different hut. Each pair was randomly assigned to one of two levels of bug abundance (30–31 or 90–91 fifth-instar nymphs of *T. infestans*); the upper bug density level was chosen because it had revealed negative density-dependent effects on domestic bug host-feeding patterns whereas the lower one did not [7]. The trial was replicated on three successive nights in the absence of any artificial source of light. Each host species was housed in a different room. Hosts were rotated among huts and between rooms every night, so that each individual host was matched with a different individual of the other host species during each of the three nights, and each individual room housed alternate host species in successive nights. Before the hosts were stationed within the huts at 8 p.m., the bugs were placed in a box with holes on the central wall at mid-day, and recovered after dismantling the movable parts of each hut on the next morning at 8 a.m. On recovery, all insects were immediately brought to the insectary, counted, scored for degree of engorgement, kept for 2 days post-recovery and then weighed (to allow them to approach the body weight plateau after eliminating the surplus of water in the bloodmeal), put in a vial labeled with a unique identifier for each bug, and then frozen at −20°C until dissection and bloodmeal identification. A subsample of 20 bugs not exposed to the hosts (control bugs) was frozen at −20°C at the same time as the recovered bugs to check whether there was any residual chicken bloodmeal in them. Given the experimental setup, we exclude the possibility that the small proportions of lost bugs escaped from the caged huts, and assume that lost bugs were most likely eaten by hosts. The proportion of blood-fed bugs is defined as the number of fed bugs (including little-fed, medium-fed and fully-fed bugs) plus bugs in the unfed nutritional class that later were ELISA-reactive to the test host species, relative to the total number of bugs examined for nutritional status; “fed” is therefore a composite category adjusted for bloodmeal reactivity among unfed bugs. The proportion of engorged bugs is defined as the sum of bugs medium fed and fully fed relative to the total number of fed bugs. The second trial, conducted in June 2006 (late fall), included three pairs of dog-cat and used the same protocol as the first trial except that hosts were stationed in the huts at 6 p.m. One replicate could not be finished properly because the cat fled away at the outset; this replicate was excluded from all calculations and analysis.

Temperature and relative humidity inside the huts were measured using data loggers (Hobo H08, Onset) inserted into the thatched roofs of both rooms and on the outside wall of a hut in the first trial, and on each of the three huts in the second trial. In the dog-chicken trial, mean internal temperatures from 8 p.m. (sunset) to 8 a.m. over the three trial nights were 22, 24 and 21°C, respectively; the mean temperature difference between rooms within a hut (dog-to-chicken) in the stated period ranged from −0.2 to +0.7°C. In the dog-cat trial, mean (minimum, maximum) internal temperatures from 6 p.m. to 8 a.m. over the three huts in each trial night were 8.8 (5.4, 13.3), 7.6 (3.7, 12.2), and 10.0°C (7.8, 14.9), respectively. The mean temperature difference between rooms within a hut (dog-to-cat) averaged over the three huts for each trial night was −0.52 (SD, 0.46), +0.09 (SD, 0.27), and −0.41°C (SD, 0.53).

### Identification of Bloodmeals

Standardization of the direct ELISA assay was based on previous procedures [33],[34] and the ELISA reagents' manufacturer manual (Kirkegaard & Perry Laboratories (KPL) Inc., Gaithersburg, MD).

#### Preparation of bloodmeal samples

Bloodmeal contents of laboratory-reared *T. infestans* nymphs fed only on goat, dog, cat, chicken and human and then frozen at −20°C were used to standardize the ELISA assay. The individual bloodmeals were prepared for testing by cutting the thorax transversally at the level of the third pair of legs, and then by expressing the bloodmeal out of the crop into a labeled vial. The bloodmeal contents were weighed, diluted from 1∶5 to 1∶50 with 0.01 *M* phosphate buffered saline (PBS), pH 7.4, and then frozen at −20°C until testing.

#### ELISA procedure

Peroxidase conjugates for identifying dog, chicken and cat blood meals obtained from KPL were reconstituted according to the manufacturer's instructions in 1∶1 of distilled water and glycerol. Bloodmeals were diluted in PBS (1∶5,000) and 50-µl volumes of the dilution were added to wells of polyvinyl chloride, mid-binding flat-shaped bottom, 96-well microtiter plates (Greiner Bio-one). Control sera were diluted 1∶60,000. The plates were covered with an acrylic plate and incubated at 37°C for 1 h, or at 4°C for 15–20 h. Well contents were aspirated and the plate was tapped against a stack of paper towels to remove excess liquid. Each well was then washed three times with PBS containing 0.01% Tween 20 (PBS-Tween 20), followed by the addition of 200 µl of the blocking buffer (BB; PBS plus 3% milk; San Regin no-fat milk) and incubated at 37°C for 1–2 h. Meanwhile, the antibody-enzyme conjugate (antihost IgG-peroxidase) was prepared by adsorbing the corresponding antisera in PBS plus 0.05% milk at 37°C for 1 h (AbC-BB). Adsorptions for each antihost IgG involved approximately 16 µl of each heterologous serum for each 8 ml of specific AbC-BB solution [33]. For the domestic hosts of *T. infestans*, we used the following adsorptions: antichicken-IgG with goat and cat sera (because no cross-reaction with other mammal sera was observed at our test conditions, and to minimize pre-incubation time); antidog-IgG with cat, chicken and human sera, and anticat-IgG with dog, human and chicken sera. Well contents were aspirated after 1 h, washed three times with PBS-Tween 20, and left at −20°C if they were scheduled for use on the following days.

For the immune reaction, 50 µl of the AbC-BB solution were added to each well and incubated at 37°C for 4 h. Well contents were aspirated again and washed three times with PBS-Tween 20. For the enzymatic reaction, 100 µl of ABTS (2,2′-azino-di[3-ethyl benzthiazoline sulfonate]) peroxidase substrate (KPL Inc.) left for 1 h at room temperature prior to utilization were added to each well and left at room temperature for 10–15 min. The reaction was stopped by adding 100 µl of SDS stop solution 1× (KPL Inc.) to each well. Absorbance at 405 nm was determined with an ELISA reader (Bio-rad 680 XR).

Each microtiter plate contained test and control samples of the target host species and four negative controls (i.e., bloodmeal contents of heterologous host species -goat, dog, cat, and chicken- in PBS). Samples were considered positive if absorbance values exceeded the maximum OD of four negative control bugs (cut-off) plus three times the standard deviation of the mean. The indeterminate zone was taken to include 10% around the cut-off. Samples were tested in duplicates, and the outcome was considered valid if the mean of both duplicates of each sample did not differ by more than 15%.

Sensitivity and specificity of the direct ELISA for the target host species was evaluated by testing bugs fed to repletion separately on a dog, cat and chicken, killed 7–30 days after feeding and held frozen at −20°C. Bloodmeal contents were weighted and diluted in PBS to 1∶5,000. A total of 44 bloodmeals on each of the three test host species was tested. The sensitivity of the direct ELISA test was 100% for all three host species. Specificity was 100% for dog and cat, and 97% for chicken.

#### Bloodmeal testing

In the dog-chicken trial, all bloodmeals from low-vector density replicates and a randomly chosen sample (>50%) of the blood-fed insects pertaining to high-vector density replicates were tested by ELISA. The latter procedure was followed because of the very large number of fed bugs available for testing, and the large preference for dogs observed. In the dog-cat trial, all bloodmeals from insects recovered were tested by ELISA (including bugs scored unfed) to allow for the possibility that bugs achieved very small bloodmeals on cats.

### Data Analysis

The data collected were entered in an Access database. Feeding indices (FI) were calculated as the ratio of the number of bugs that fed on a given host species X to the number of bugs that fed on the matched host species Y (whether or not the bugs that fed on X fed on Y and vice versa). As only one host of each host species was present we did not need to correct for the number of hosts [35].

Four related measures of blood gain by the bugs were used: i) feeding success, a binary variable measuring the likelihood of blood-feeding on any one or on both host species inside the hut (i.e., overall feeding success: fed bugs relative to the number of bugs recovered alive or dead), or on a specific host species as determined by ELISA (i.e., host choice); ii) engorgement (a binary variable including medium-fed and fully-fed bugs: engorged bugs relative to the number of fed bugs); iii) nutritional status (a categorical variable with four levels), and iv) post-exposure bug weight (a continuous variable, measured two days after host exposure). Engorgement and post-exposure bug weight measure the amount of blood imbibed overall or on a given host species. Exact 95% confidence intervals (95% CI) for mean vital rates (i.e., binary variables) were based on the binomial distribution.

The effect size on several binary response variables was estimated by fitting random-effects logistic regression models clustered by hut to the data using the command xtlogit in Stata 9.1 [36]. The use of random-effects models addresses the fact that insects within a hut roughly share the same environment and other undetermined characteristics that may create dependencies between responses within the same cluster of observations. We tested for significant (*P*<0.05) effects of trial (dog-chicken trial = 1; dog-cat trial = 2), vector density (two levels) and occasion (three levels) on several vital rates: bug recovery (including both dead and alive bugs relative to the number of released bugs); bug loss and mortality (missing and dead bugs relative to the number of released bugs, respectively); overall feeding success and engorgement, as defined above. An interaction term between vector density and occasion was added to each main-effects model. Host-feeding choices were analyzed by conditional (fixed-effects) logistic regression using McFadden's choice model with the command clogit and robust standard errors. These analyses only included unmixed host choices (i.e., dog, other) in each trial because bugs with mixed or no bloodmeal could not be considered for this analysis. To examine whether host choices were modified by vector density levels, occasion and individual dog, interaction terms were added to each of the models. Alternatively, exact binomial tests were used to test for differences between host choices in each replicate relative to the null hypothesis of no selective host choice. Random-effects multiple linear regression with the command xtreg was used to test for significant effects on post-exposure bug weight of vector density, host blood source (unmixed), nutritional status and occasion. Interaction terms were added one by one to the model with main effects and retained in the final model if *P*<0.1. When the response variable was nutritional status, multinomial logit models were used.

## Results

Of 1,622 triatomine bugs released in both trials, 1,536 (94.7%) were recovered and examined for nutritional status (Table 1). Random-effects logistic regression showed that the overall loss rate of bugs was significantly higher in the dog-chicken trial (6.8%) conducted in summer than in the dog-cat trial (2.4%) run in late fall (OR = 0.32, 95% CI, 0.11–0.96, *P* = 0.042), but the reverse happened with the observed mortality rate (0.3% vs 3.3%, respectively; OR = 12.23, 95% CI, 3.56–41.98, *P*<0.001), with no significant occasion effects in both cases. Most of the dead bugs recovered were unfed (17 of 21) and had very low weight. Significantly more bugs blood-fed (98.7%) in the dog-chicken trial than in the dog-cat trial (71.4%; OR = 0.031, 95% CI, 0.016–0.058, *P*<0.001), but among the fed bugs, engorgement status did not differ between trials (46.8% vs 44.4%, respectively, OR = 0.96; 95% CI, 0.68–1.36, *P*>0.8). Vector density adjusted for occasion effects did not modify significantly any of the vital rates in both trials (Table 1).

**Table 1:** Mean vital rates (lost, dead, fed, and engorged) of fifth-instar nymphs of *T. infestans* in two host choice experiments.

Trial	Day	No. of replicates	No. of bugs released	Percentage of bugs			
				lost	dead	fed	engorged
Dog-chicken	1	6	360	8.1	0.0	98.5	48.2
	2	6	361	5.3	0.0	99.4	47.1
	3	6	360	6.9	0.8	98.2	45.3
	Sub-total	18	1081	6.8	0.3	98.7	46.8
OR for vector density effects					0.63	0.26	1.13
95% confidence interval					0.05–7.57	0.03–2.11	0.79–1.62
Dog-cat	1	2	120	4.2	5.0	64.0	39.7
	2	3	211	1.4	3.8	64.1	52.3
	3	3	210	2.4	1.9	82.8	40.2
	Sub-total	8	541	2.4	3.3	71.4	44.4
Total		26	1622	5.3	1.3	89.1	46.2
OR for vector density effects				0.34	3.85	0.62	0.55
95% confidence interval				0.07–1.74	0.50–29.62	0.35–1.08	0.30–1.00

**Figure 1:**
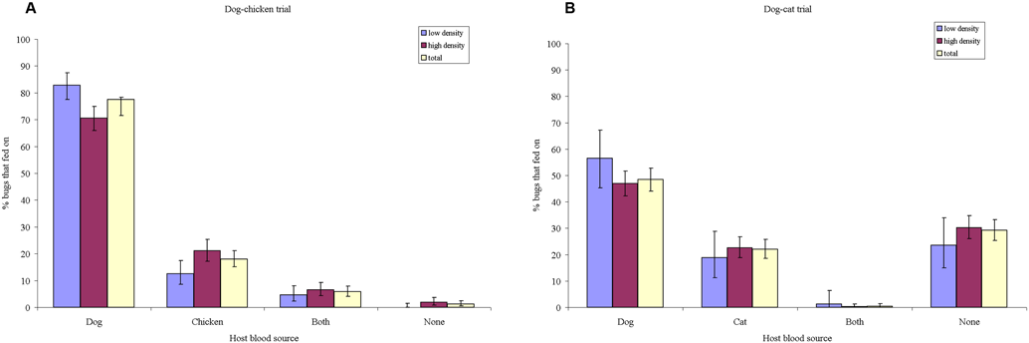
Host choice and vector density-dependence. Mean percentage of *T. infestans* fifth-instar nymphs that fed on dogs only, on the alternative host only, on both host species, or on none of them according to vector density level in two host choice trials. A, dog-chicken trial; B, dog-cat trial. Bars are 95% confidence limits.

**Figure 2:**
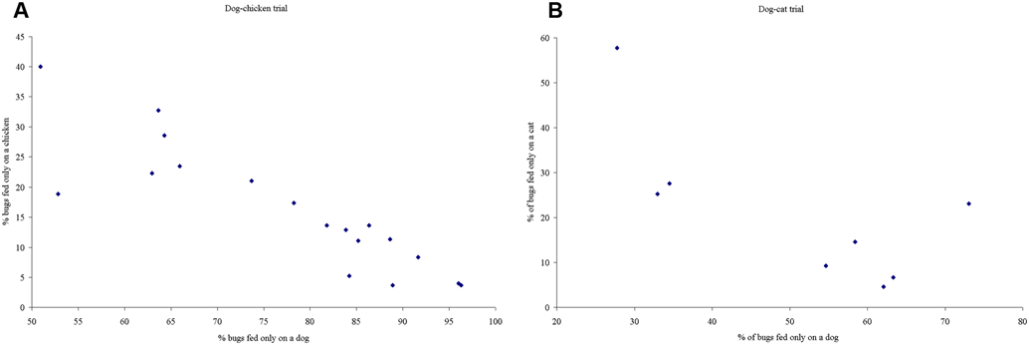
Relative host choices per hut. Relationship between the proportion of bugs that fed only on dogs and only on chickens (A) or cats (B).

In the dog-chicken trial, we observed that most of the bugs were recovered from the thatched roof of the dog's room, followed by the adobe bricks in the mid-wall; the fewer bugs recovered from the chicken's room were in the thatched roof. Of all the bugs with identified bloodmeals, 81.8% had feedings on dogs and 24.0% on chickens. The dog-to-chicken mean feeding index was 7.0 (95% CI, 3.7–10.3). The total mean percentage of insects that fed on dogs only (75.0%, 95% CI, 71.5–78.3%) was significantly higher than that on chickens only (18.0%, 95% CI, 15.1–21.1%) (Fig. 1A). Both feeding choices were highly significantly correlated (*r* = 0.88, *P*<0.001) at each hut (Fig. 2A). Only 5.8% (95% CI, 4.1–7.9%) of bugs fed on both hosts, and 1.2% (95% CI, 0.5–2.4%) on none. Conditional logistic regression showed that dogs were highly preferred to chickens (OR = 11.2; 95% CI, 6.2–20.1, *P*<0.001) and high vector density significantly reduced feedings on dogs (OR = 0.51; 95% CI, 0.32–0.82, *P* = 0.005), with significantly reduced dog choice at occasion 2 (OR = 0.37, 95% CI, 0.22–0.62, *P*<0.001). Feedings on dogs were homogeneous among individual dogs (*P*>0.1). When each replicate was taken separately, dogs were significantly preferred over chickens in 16 of 18 replicates (binomial test, *P*≤0.001 in 13 replicates and *P*<0.05 in 3 replicates; one trial was marginally significant, *P* = 0.06, and one not significant). Eighteen (47%) of the 38 mixed bloodmeals recorded were from a single dog. None of the 20 control bugs not exposed to the hosts were positive for chicken bloodmeal.

In the dog-cat trial, the apparent dispersion pattern of bugs on recovery was more mixed among days; only in one day were most of the bugs located in the dog's thatched roof. Of the bugs with identified bloodmeals, 69.0% had feedings on dogs and 31.6% on cats. The dog-to-cat mean feeding index was 4.8 (95% CI, 2.7–6.9). Significantly more bugs blood-fed on dogs only (48.5%, 95% CI, 44.1–52.8%) than on cats only (22.0%, 95% CI, 18.5–25.7%); both indices were highly correlated (*r* = 0.71, *P*<0.001) though the relation was even stronger in the dog-chicken trial (Figs. 1B and 2B). Only 0.4% (95% CI, 0.05–1.4%) of bugs fed on both hosts, and no feeding was detected in 29.2% (95% CI, 25.3–33.2%). Conditional logistic regression showed that dogs were significantly preferred to cats (OR = 7.8; 95% CI, 1.7–35.8, *P*<0.001) and vector density reduced significantly the likelihood of feeding on dogs (OR = 0.09; 95% CI, 0.01–0.66, *P* = 0.018), with significantly increased dog choice at occasion 2 (OR = 8.9, 95% CI, 1.6–48.7, *P* = 0.012). Heterogeneous feeding rates on individual dogs 3 (OR = 6.5, 95% CI, 2.6–11.1, *P*<0.001) and 4 (OR = 0.24, 95% CI, 0.07–0.84, *P* = 0.025) relative to dog 2 were recorded. Bugs significantly preferred the dog in five replicates (*P*≤0.001 in four replicates, *P*<0.02 in one) and the cat only in one replicate (*P*<0.001), whereas no significant differences were found in two replicates (*P*>0.2). Two of the cats frequently allowed the bugs to blood-feed on them though with large variations between nights. In the excluded replicate that had no cat, 93% of the released bugs were recovered and 79% of them were fed on dog only, with no feeding on cat detected.

**Figure 3:**
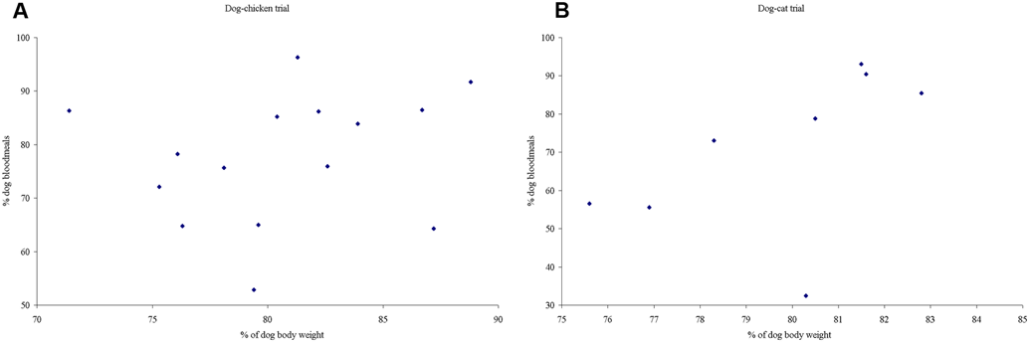
Host choice and relative host body size. Relationship between host-feeding preferences of *T. infestans* fifth-instar nymphs and the percentage of dog body weight in each matched pair of hosts in two host choice trials. A, dog-chicken trial. B, dog-cat trial.

The relationship between proportional host body weight and host-feeding preferences in both trials is shown in Fig. 3. Two different patterns were obtained. The proportion of bugs that fed on dogs and proportional dog body weight were unrelated in the dog-chicken trial, whereas a significant relationship was found in the dog-cat trial (OR = 1.21; 95% CI, 1.03–1.44, *P* = 0.022) where exclusion of an outlier value gave a stronger relationship (OR = 1.32; 95% CI, 1.20–1.45, *P*<0.001).

Table 2 shows the relation between bug nutritional status on recovery of live and dead bugs, post-exposure mean bug weight, and bloodmeal source in both trials. Before exposure to hosts, the distributions of bug weight in the dog-chicken trial (mean, 61.9 mg; 95% CI, 60.3–63.5) and in the dog-cat trial (mean, 59.3 mg; 95% CI, 56.6–62.0) were not significantly different (Anova, *F* = 2.80, df = 340, *P* = 0.095). Post-exposure mean bug weight in the dog-chicken trial (238.1 mg) was significantly higher than in the dog-cat trial (136.8 mg) (Anova, *F* = 432.3, df = 1,503 and 1, *P*<0.001), and steadily and significantly increased with nutritional status class in both trials (Anova, dog-chicken: *R^2^* = 0.61, *F* = 515.6, df = 973 and 3, *P*<0.001; dog-cat: *R^2^* = 0.59, *F* = 253.0, df = 524 and 3, *P*<0.001). In total, 1,160 bugs were tested by ELISA and bloodmeals from 1,023 bugs were identified. The percentage of bugs with dog bloodmeal only varied marginally from 74.8% to 81.8% among nutritional classes in the dog-chicken trial (χ^2^ = 6.60, df = 3, *P* = 0.086), but increased significantly from 35.0% to 62.5–76.0% in the dog-cat trial (χ^2^ = 16.1, df = 3, *P*<0.001). The fraction of bugs with mixed meals on dogs and chickens steadily increased with nutritional status up to 12.1% in the fully-fed bugs. More bugs in the unfed nutritional class were ELISA-reactive in the dog-chicken trial (68.7% of 16) than in the dog-cat trial (15.9% of 126) (Fisher's exact test, *P*<0.001). Most bugs classified as little-fed in the dog-cat trial and not reactive to dog or cat by ELISA had residual chicken bloodmeals taken in the insectary three months before.

**Table 2:** Association between bug nutritional status after exposure, mean bug weight two days after host exposure and host-blood source of fifth-instar nymphs of *T. infestans* in two host choice experiments. 95% CI, 95% confidence interval.

Trial	Nutritional state class	No. of bugs examined	Weight (mg)		No. of bugs reactive (tested)	Percentage of bugs fed on		
			Mean	95% CI		dog	other	mixed
Dog-chicken	Unfed	22	53.3	46.7–59.9	11 (16)	81.8	18.2	0.0
	Little fed	520	178.7	173.6–183.9	341 (343)	74.8	22.0	3.2
	Medium fed	373	301.3	295.4–307.3	239 (239)	76.6	15.1	8.4
	Fully fed	93	353.8	342.2–365.3	58 (58)	79.3	8.6	12.1
	Sub-total	1008	238.1	232.3–243.9	649 (656)	76.0	18.2	5.9
Dog-cat	Unfed	145	57.9	54.8–61.0	20 (126)	35.0	65.0	0.0
	Little fed	213	123.6	117.1–130.2	188 (212)	66.5	33.5	0.0
	Medium fed	154	210.2	197.5–222.9	150 (150)	76.0	22.7	1.3
	Fully fed	16	319.9	283.7–357.1	16 (16)	62.5	37.5	0.0
	Sub-total	528	136.8	129.4–144.1	374 (504)	68.5	31.0	0.5

**Figure 4:**
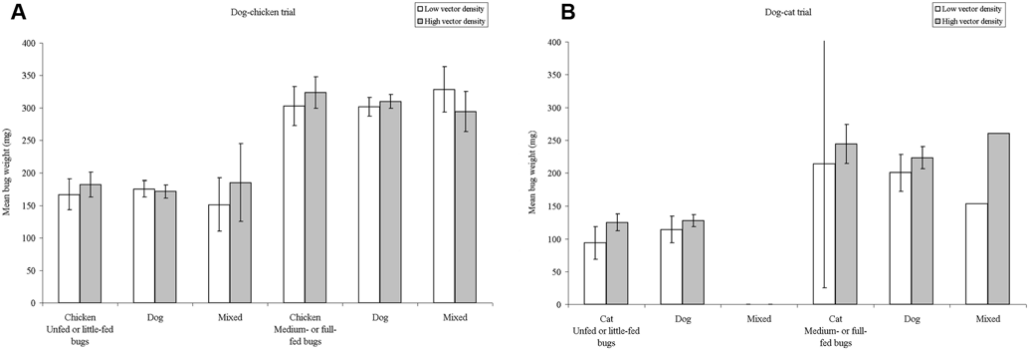
Engorgement, vector density and host choice. Relationship between mean weight of fifth-instar nymphs of *T. infestans* two days after host exposure and host-blood source, vector density, and post-exposure engorgement status in two host choice experiments. A, dog-chicken trial. B, dog-cat trial. Bars are 95% confidence limits.

The post-exposure engorged status and mean bug weight of *T. infestans* according to vector density and individual host blood source are shown in Table 3 and Fig. 4, respectively. In the dog-chicken trial, the percentage of blood-engorged bugs was higher if the bug fed on dog only (43.4–48.5%) rather than only on chicken (34.1–36.7%), whereas bugs with mixed bloodmeals were more frequently engorged (70.4–72.7%) than those with unmixed meals (Table 3). Relative to little-fed bugs (unfed bugs were rare), the relative risk ratio (RRR) of a bug being medium-fed (RRR = 1.62, 95% CI, 1.02–2.57, *P* = 0.039) or fully-fed (RRR = 2.1, 95% CI, 0.8–5.3, *P* = 0.14) was significantly higher if the bug had fed on a dog only, after adjusting for significant occasion effects (*P*<0.001) and non-significant (*P*>0.4) vector density effects (n = 600, χ^2^ = 44.3, *P*<0.001, AIC = 1059.2, df = 10). All two-way interaction terms were not significant. Post-exposure mean bug weight varied significantly (*P*<0.001) with nutritional status and its interaction with occasion but not with vector density or host blood source (*P*>0.6) (*R* ^2^ = 0.606, n = 583, *P*<0.001) (Fig. 4A). In the dog-cat trial, the dog-fed bugs engorged significantly more than the cat-fed bugs at lower vector densities (64.6% vs 18.8%, respectively), but there were smaller differences at higher levels of infestation (44.7% vs 37.0%, respectively) (Table 3). When compared to unfed bugs, the relative risk ratio of a bug being little-fed (RRR = 4.7, 95% CI, 1.8–12.4, *P* = 0.002), medium-fed (RRR = 7.2, 95% CI, 2.6–19.5, *P*<0.001) or fully-fed (RRR = 3.8, 95% CI, 0.98–15.1, *P* = 0.053) increased significantly if the bug had a feeding on dog only, after adjusting for significant occasion effects (*P*<0.02) and marginal effects (*P* = 0.056) of vector density (n = 372, χ^2^ = 31.9, *P*<0.001, AIC = 745.3, df = 15). Post-exposure mean bug weight (log-transformed to normalize the distribution) was significantly modified by host blood source (*P* = 0.022), vector density (positively, *P* = 0.008), nutritional status (positively, *P*<0.001) and occasion (*P*<0.001) (Fig. 4B). Addition of interaction terms revealed significant effects (*P*<0.03) between occasion and vector density or nutritional status (n = 372, *R^2^* = 0.75, *P*<0.001).

**Table 3:** Association between nutritional state after exposure, host-blood source and vector density of fifth-instar nymphs of *T. infestans* in two host choice experiments.

Trial	Vector density	Host blood source	No. of bugs examined	Percentage of bugs	
				engorged	fully fed
Dog-chicken	Low	Dog	198	43.4	8.6
		Chicken	30	36.7	3.3
		Mixed	11	72.7	36.4
	High	Dog	295	48.5	9.8
		Chicken	88	34.1	4.5
		Mixed	27	70.4	11.1
Dog-cat	Low	Dog	48	64.6	6.3
		Cat	16	18.8	0.0
		Mixed	1	100.0	0.0
	High	Dog	208	44.7	3.4
		Cat	100	37.0	6.0
		Mixed	1	100.0	0.0

## Discussion

Results of this study strongly refute the hypothesis that *T. infestans* prefers to blood-feed on chickens rather than on dogs. *T. infestans* consistently preferred dogs and engorged significantly more on dogs than on chickens or cats. Although chickens were relatively more often selected than dogs in domestic bug collections during the hot season [7] and in overnight experiments [19], in our semi-natural experiments *T. infestans* consistently preferred dogs to chickens despite: i) the bugs having had prior feeding experience on chickens in the insectary; ii) chickens having higher average body temperature (41–42°C) than dogs or cats (38–39°C), and iii) *T. infestans* blood-feeding more easily on pigeons or chickens than on mice and probably on other mammals in general (depending on the exact biting site) because bird blood lacks platelets and some coagulation factors and therefore is easier to imbibe [37]. Strict quality control methods practically exclude the chance of ‘false negative’ bloodmeal results, and restricted unspecific results to <3% (only in the case of chicken bloodmeals).

Several non-mutually exclusive factors may contribute to explain the feeding preference of *T. infestans* for dogs rather than chickens or cats: i) larger body size and surface; ii) greater attractiveness mediated by dog-specific cues or odors, yet to be identified and demonstrated, and iii) less defensive reactions to bug bites. On average, the study dogs had 4.5 times (range, 3.7–5.4) more biomass than the study chickens and 4.0 times (range, 2.8–5.2) more than the cats, thereby producing more total heat, moisture and carbon dioxide – known bug attractants. Yet the quantitative preference for feeding on dogs only was directly and significantly related to relative dog biomass in the comparison with cats but not with chickens. A laboratory-based comparison of host-feeding choices between chickens and guinea pigs also showed no direct relationship with host biomass [18]. It appears that relative host biomass would play a lesser role in determining feeding choices of *T. infestans* between two animal host species than in the case of mosquitoes and humans [38], possibly because of differences in behavior between host species.

The observed host-preference for and higher degree of blood-engorgement on dogs may be explained by higher host attractiveness and less effective host defensive behavior, factors that have been found to be inversely associated in other insect vectors, as predicted by evolutionary theory [39]. Our study provides the first evidence showing that the dog-fed bugs achieved a significantly larger relative degree of engorgement than the chicken- or cat-fed bugs, and higher post-exposure bug weight than the cat-fed bugs. The apparent inconsistency between blood-engorgement status and bug weight at two days after exposure in the dog-chicken trial may be attributed to large differences between chicken and dog blood in water and DNA content that modify the rate of bug diuresis and blood digestion depending on the type of blood imbibed. In field studies, both dogs and chickens were taken to be more tolerant to domestic *T. infestans* than cats because (i) dog and chicken bloodmeals were more often unmixed than cat bloodmeals (i.e., repeated or continuous feedings on cats were much less likely than on other hosts) [15],[20],[21], and (ii) the likelihood of feeding on dogs was positively density-dependent after adjusting for the observed number of each host species at each household [7]. However, the same mixed host-selection pattern could have been caused by equally tolerant hosts having radically different exposure patterns (i.e., stable for dogs and chickens vs unstable for cats) as discussed below. Unlike the study dogs, rural dogs in some endemic areas in northern Argentina frequently were larger, undernourished (sometimes severely), had lower hematocrits and suffered from other diseases [40]. This combination of factors probably made these rural dogs less responsive and allowed bugs to engorge more easily. Our current results show that the bugs' apparent preference for dogs still holds when the dogs were well-fed and healthy. The direct association between having fed on a dog and bug engorgement level or post-exposure weight demonstrates that on average, dogs were more tolerant to bug bites than cats or chickens. The host choice experiment results are therefore consistent with host-feeding patterns in real-life settings.

Mixed bloodmeals on dogs and chickens obtained within the same night most likely stemmed from interrupted, incomplete feeds caused by host-defensive behavior, and cannot be attributed to previous meals at earlier instars in the insectary (as shown by control bugs). Although our experiment does not allow to distinguish the host on which the bugs with mixed meals engorged first, bugs probably switched from the more defensive to the more tolerant or preferred host (i.e., dogs) to satiate, as suggested by mosquito-bird studies [2],[41]. Overnight videotaping of a chicken exposed to the bugs in one of our huts showed strong anti-feeding activity expressed in stamping and other defensive reactions (M.C. Cecere, unpublished observations). These anti-bug behaviors were frequently reported by local villagers when infestations associated with brooding hens were large. Similarly, after a two-week exposure to two matched pairs of hosts, *T. infestans* bugs were more frequently found spatially associated with guinea pigs than with chickens [18]. In our experimental setup, both chickens and dogs were sources of bug mortality [27],[31] whose relative magnitude is unclear. In addition, strong host heterogeneity in response to bug activity reflected in that individual dogs differed largely in the likelihood with which they provided a bloodmeal relative to cats, not to chickens; their contribution to mixed bloodmeals relative to chickens, and background history of eating bugs. When chickens were fed upon, they apparently allowed as large a bloodmeal as dogs.

Both trials displayed significant vector density-dependent effects on individual host choice, blood-engorgement and/or post-exposure bug weight (not on overall feeding success or other vital rates measured at the hut aggregate level), though such effects were more complex than expected. In laboratory settings several triatomine bug species frequently showed negative density-dependent engorgement rates on non-anesthetized, unrestrained, small hosts including mice, hamsters, guinea pigs, small chickens and pigeons [13], [17], [30], [42]–[44]. No such experiment including dogs or cats has been reported. In our study, negative density-dependent dog choice implied that host shifts occurred more frequently at higher vector densities, as predicted by optimal foraging models [2]. However, both engorgement and post-exposure bug weight were not modified by vector density in the dog-chicken trial, whereas bugs in the dog-cat trial achieved higher post-exposure engorgement and weight at higher vector densities over occasions, more so if they had fed on a dog. This may be due to the development of increasing host tolerance to increasing bug bites over successive occasions, as observed before [42], or to a relative increase in mean temperature from 7.6 to 10°C between occasion 2 and 3. Although the effects of vector density on bloodmeal size were highly significant, they were not particularly intense (Fig. 4B). However, the tested vector densities (and inferred attack rates) in the experiments were rather low compared with intense domestic infestations sometimes reaching a few thousand domestic *T. infestans* bugs per house. Density-dependent effects need to be investigated at a wider range of densities for a full description of the functional relationship between vector density and host-feeding success or bloodmeal size.

Temperatures were nearly homogeneous within each trial but differed largely between trials. The lower bug feeding success and post-exposure weight in the dog-cat trial conducted in late fall is most likely explained by a sharp fall-off in temperature relative to the dog-chicken trial conducted in summer, rather than by the presence of less suitable hosts (i.e., cats). Low temperatures also increased overnight mortality rate; probably reduced bug mobility, exposure to the hosts and losses due to predation, and reduced diuresis regardless of bloodmeal size [45]. We chose to complete the experiments in the fall because *T. infestans* populations blood-fed and maintained a high nutritional status during the cold season in the same study setting ([31], unpublished data), unlike in other places [46],[47]. Given that the spontaneous locomotory activity of *T. infestans* was strongly reduced below 18°C in laboratory experiments [48], it is remarkable that most of the bugs succeeded in blood-feeding at hut internal temperatures averaging 7.6–10.0°C (range, 3.7–14.9°C) during the host exposure period, or at 10.8–12.4°C during the first two hours after sunset when most bugs engage in host-seeking activities. Both the success and intake rate of *T. infestans* from an artificial feeding apparatus increased linearly with blood temperature between 6 and 35°C, but this occurred after an initial stimulation with a heated steel plate at 35°C [49]. In our study, the relationship between temperature and bug activity may have been severely modified by the use of starved bugs and the size and confined features of the huts, implying increased motivation and suitable conditions for host-seeking activities. A reappraisal of the association between the feeding frequency of *T. infestans* and temperature under different bug physiological states and field conditions is warranted.

Our experiments quantified host-feeding preferences in replicated trials using mongrel animals similar to those owned by rural villagers (though our dogs were older), and were conducted at more natural conditions than laboratory-based trials. Unlike field-based host-feeding patterns, host choices were not confounded by nonrandom temperature variations between house structures or huts over time; unequal host availability or accessibility, or by stationing host species in fixed rooms within each hut. Therefore, the patterns revealed by the host choice experiment may be taken as the base case against which to compare the more complex field data for additional inferences on the effects of host availability or accessibility.

The observed preference for dogs rather than chickens contradicts field data analyzed by household and bug collection habitat [7]. The dog-to-chicken median feeding index (0.4; interquartile range Q_1_–Q_3_, 0.1–1.6) of domestic *T. infestans* in spring-summer tended to favor chickens and was highly variable among households, whereas in the host choice trial the mean FI consistently favored dogs (7.0) and was 12-fold larger. Similarly, the median dog-to-cat FI of domestic *T. infestans* across seasons favored dogs (1.7; Q_1_–Q_3_, 0.5–4.1; calculated from 24 dog- and cat-owning households with dog and cat bloodmeals from the Appendix in [7]), but in the host choice trial the mean FI was nearly three-fold larger (4.8). Therefore, on a per capita basis and assuming host availability at observed values, *T. infestans* blood-fed on dogs much more often than on cats in the experimental host choice trial than in the field by a factor of 2.8 (i.e., feedings on cats occurred relatively more often in the field), with large variability in both settings.

Demographic and behavior patterns of domestic animals may explain the observed discrepancies between field and experimental host choice data. In rural villages, most of the between-house variability in chicken feedings may be attributed to the seasonal brooding curve of fowls, which peaked in mid-spring and decreased during the hot summer months, and to heterogeneous fowl breeding practices among households [50]. Domestic *T. infestans* took advantage of the constant indoor location of brooding hens during extended periods (put indoors for protection against predators), and of their apparent less responsive behavior during brooding. With respect to dogs and cats, both populations greatly differed in abundance (3∶1) and were approximately stationary in size, with high annual turnover rates (>30%) and predominance of males (range, 67–85%) in northern Argentina [51]. Although 50%–68% of the dogs and cats were reported to sleep in domestic sites (albeit with undetermined frequency), they were not restrained or confined and many roamed freely within and around the village to forage for all or part of their food. Dogs were sometimes reported to free-range in small packs at night (probably in oestrus groups with several males), whereas 56% of the cats were reported to stray in the forest to hunt. These qualitative features appear to be common in resource-poor rural settings in northern Argentina where Chagas disease is hyperendemic, and where there is little demarcation of property lines. In other rural locations, adult male dogs and cats have a larger home range and longer duration of activity than females; dogs display a particularly marked crepuscular free-ranging behavior with two peaks of daily activity [52],[53]. These peaks of activity mostly coincide with the main host-seeking periods of *T. infestans* bugs, and would reduce host availability and the likelihood of dog-vector encounter at such periods. We infer that current domestic bug host-feeding patterns reflect limited, heterogeneous dog exposures, and there would be ample room for increased feedings on dogs with increased dog exposure.

The long-standing controversy on the role of cats in the domestic transmission of *T. cruzi* infection revolved around the notion that their well-known nocturnal activity pattern and apparent intolerance to bug bites would reduce the likelihood that domestic bugs blood-fed on cats [29]. However, several studies showed that domestic *T. infestans* and other triatomine species blood-fed on cats as frequently as or more than on dogs [7],[20], a pattern that also emerged in our host choice experiment. To the apparent inconsistencies between different pieces of evidence already discussed [21], we add geographic variations in pet ownership and keeping practices, and heterogeneities in host behavior and exposure between demographic subgroups of cats (by age, sex and reproductive status). In our rural study area, kittens and young pups were frequently kept indoors all day long. Cats avoided exposure to hot weather by resting indoor in the cooler, dark mud-and-thatch houses during the daytime. Under such conditions, residents of highly infested houses sometimes reported to be attacked by *T. infestans* nymphs when resting on the floor indoors at noon (unpubl. observations). Age- and sex-dependent host activity patterns modify host exposure to domestic vectors, which combined with individual host heterogeneities and other factors, explain the large variability in feeding patterns observed between households and studies.

Some aspects of our study design limit the interpretation of results. Lack of direct observations on host attractiveness and defensive behavior limits the interpretation of the observed host-feeding preferences in terms of underlying mechanisms. Although both trials had the same initial density of bugs per hut, different temperatures affected attack, feeding and other vital rates; therefore, we refrained from establishing quantitative comparisons between trials and focused on within-trial outcomes. Bloodmeal size was not recorded directly, but nutritional status and post-exposure weight were strongly positively correlated; both may be considered valid surrogate indices of bloodmeal size given that pre-exposure bug weight was very similar between trials. Post-exposure bug weight should be increased roughly by 50% to estimate the actual weight immediately after feeding because of the rapid loss of excess water during the first day or two post-feeding depending on temperature. This rough calculation and comparison with laboratory-based estimates of bloodmeal size suggests that most of the bugs blood-fed to repletion in the summer trial. Because only the study dogs had been bitten by *T. infestans* before the experiments, differences in their background experience with bugs might modify the bugs' preference for dogs. This effect may not be too serious, considering that naïve chickens have an innate, stable tendency toward tolerating or defending from triatomine bites even after a rest time between exposures [42]. The individual study dogs also expressed some idiosyncratic behaviors against bugs over time. Despite the fact that the study dogs had been exposed to *T. infestans* bites six months before the first trial (i.e., were presumably immunized against bug salivary antigens), most of the dog-fed bugs blood-engorged close to repletion. Therefore, it seems very unlikely that the dogs' immune response reduced substantively vector host-feeding success or engorgement. Immune reactions to bug saliva on the skin of previously sensitized chickens facilitated vector blood-feeding [54] but we have not observed such skin reactions in dogs. It is unlikely that previous dog-bug contacts may have influenced the various steps involved in the host-selection process prior to engorgement; whether the degree of bug engorgement was modified by prior exposure of dogs remains to be determined. Unlike chickens found indoors in rural villages, the study hens were not brooding and their behavior in response to bugs may be potentially different.

### Implications for Disease Control and Modeling

Increased host tolerance implies increased residence and feeding times on the host, which in turn will increase fitness by increasing the overall rate at which blood is obtained, eggs are produced, and survival per feeding attempt [55]. The nutritional quality of blood may differ substantively between host species of *R. prolixus* [56], with chicken blood having half the hematocrit than mammals and much lower hemoglobin or plasma protein than dogs [57]. Therefore, the aggregate fitness implications of host choices remain to be established. Of note, the host bloodmeal choice variable includes a survival component because it was measured on recovered, fed bugs.

Because bloodmeal size increases the probabilities of *T. infestans* emitting dejecta sooner [58], ingesting trypanosomes and becoming infected [21], it follows that preferred, tolerant hosts such as dogs will seriously increase transmission rates relative to other domestic hosts. By virtue of allowing larger bloodmeals, the likelihood of dogs being repeatedly contaminated with bug feces and eventually superinfected with various parasite strains would be increased. The large frequency of unmixed dog bloodmeals shown by *T. infestans* in some field locations further suggests that a strong, stable link between individual dogs or groups of dogs and groups of bugs occurs in some households, thereby increasing transmission of *T. cruzi* back and forth from dogs to bugs and creating a transient partial refuge for other host species (a zooprophylactic effect). In most households, however, the frequency of mixed bloodmeals on dogs is high during spring-summer, and because domestic host species and bugs are more connected the flux of parasites between them is enhanced. Selective host choice amplified by a greater feeding success on diseased or infected hosts will increase the basic reproduction number of *T. cruzi* (though with possibly depressed prevalence and incidence as the outbreak follows through) compared with the base case represented by homogeneous contact rates [11],[12],[59]. An increase in dog or cat availability or accessibility in domestic areas will increase the rate of bug feeding on them which in turn will exert non-linear effects on *R*_0_ through the squared biting rate term. When the proportion of insects feeding on a given host species (i.e., humans) varies with the relative abundance of non-human (i.e., dogs, cats, chickens) and human hosts and with the ratio of vectors to hosts, as our studies have shown, the relationship between *R*_0_ and host blood indices is predicted to be strongly non-linear [2]. This implies that different tactics that seek to reduce vector abundance will exert very different impacts on parasite transmission depending on the exact relationship between *R*_0_ and the vector-to-host ratio. The empirical evidence further supports the prediction that removal of dogs from bedroom areas will strongly decrease domestic bug population size, transmission rates and human incidence of infection [22].

Heterogeneities in vector feeding rates and in host exposure and infection will tend to create ‘hot’ and ‘cold’ spots of transmission, which can be used to target more accurately and efficiently host species and individuals accounting for most of the risk. Application of pyrethroid-impregnated dog collars, causing reduced repellency but increased bug mortality for extended periods [27], are predicted to strongly reduce domestic bug population size and transmission rates. The various layers of heterogeneity involving dogs in rural endemic areas, including household aggregation of infection, infectiousness to bugs and exposure patterns [21],[51], can be used when designing control measures. For increased impact, collars or other similar tools should be preferentially applied to those dogs that are infected with *T. cruzi* and/or highly infectious to bugs and that are also closely associated with domestic sites (e.g., pups, females in reproductive state or restrained dogs). Such dogs can be turned into baited lethal traps, though a thorough cost-effectiveness assessment of such tactics is needed before large-scale field application. Other possible applications are to use dogs as baited sentinels of bug presence through the use of its immune response to salivary antigens for serologic surveillance during a bug elimination campaign [60] and as sentinels of parasite transmission [51].

## Supporting Information

Alternative Language Abstract S1 Translation of the abstract into Spanish by Ricardo E. Gürtler
(0.02 MB DOC)
