# Supplementary material for: Strong Host-Feeding Preferences of the Vector Triatoma infestans Modified by Vector Density: Implications for the Epidemiology of Chagas Disease
Source: PLoS Negl Trop Dis. 2009 May 26;3(5):e447. doi: 10.1371/journal.pntd.0000447 (PMC2682203; doi:10.1371/journal.pntd.0000447)
Supplement: Alternative Language Abstract S1 — Translation of the abstract into Spanish by Ricardo E. Gürtler (0.02 MB DOC) [file pntd.0000447.s001.doc]

**Resumen**

***Antecedes:*** La comprensión de los factores que afectan las preferencia de alimentación de los insectos triatominos que transmiten el Mal de Chagas es crucial para estimar riesgos de transmisión y predecir el efecto de tácticas de control dirigidas a los animales domésticos. Investigamos si el vector *Triatoma infestans* prefiere alimentarse sobre perros en vez de gallinas y sobre perros en vez de gatos cuando todos estos no se hallan inmovilizados, y si la densidad de insectos modifica la elección de hospedadores, el éxito de alimentación, el tamaño de la ingesta sanguínea y otras tasas vitales bajo condiciones climáticas naturales.

***Metodología:*** Se realizaron dos experimentos de elección de hospedadores en ranchos experimentales pequeños enjaulados que contaban con dos habitaciones entre las cuales los insectos se podían mover libremente. Se asignaron al azar seis pares de perro-gallina y tres pares de perro-gato a dos niveles de abundancia de vectores, y se expuso a los pares de hospedadores a insectos no alimentados durante tres noches consecutivas. Se investigaron las fuentes de alimentación sanguínea de 1.160 insectos recuperados posteriormente mediante un ensayo inmunoenzimático directo.

***Principales Hallazgos:*** Un análisis de regresión logística condicional mostró que los insectos prefirieron alimentarse sobre perro que sobre gallinas o gatos, y que la densidad del vector modificó las elecciones de alimentación sobre los hospedadores. El riesgo relativo de que un vector se hubiera llenado de sangre se incrementó significativamente cuando se alimentó solo sobre perro en vez de solo sobre gallina o gato. Los insectos alcanzaron un mayor peso post-exposición a altas densidades de vectores y en ocasiones sucesivas, y más aún si se habían alimentado sobre perro en vez de gato.

***Conclusiones:*** Estos resultados refutan fuertemente la hipótesis que *T. infestans* prefiere alimentarse sobre gallinas en vez de perros. Si se produce un incremento en la disponibilidad o accesibilidad de perros o gatos en las áreas domésticas, se incrementaría la tasa de alimentación de los vectores sobre ellos, lo cual a su vez ejercería fuertes efectos no lineales sobre *R_0_*. Combinada con heterogeneidades entre perros en su exposición, infección e infectividad, la fuerte preferencia de *T. infestans* por los perros puede ser explotada tratándolos en general, y más específicamente a los individuos que contribuyen la mayor parte del riesgo de transmisión, con lociones tópicas o collares impregnados con insecticidas para tornarlos en trampas cebadas letales, o aprovechándolos como centinelas de la transmisión o de la infestación mediante su respuesta inmune al parásito o a antígenos salivales del vector.
